# Impact of the Supervisor Feedback Environment on Creative Performance: A Moderated Mediation Model

**DOI:** 10.3389/fpsyg.2017.00256

**Published:** 2017-02-22

**Authors:** Jian Zhang, Zhenxing Gong, Shuangyu Zhang, Yujia Zhao

**Affiliations:** ^1^Donlinks School of Economics and Management, University of Science and Technology BeijingBeijing, China; ^2^Laboratory of Talent Evaluation of Land and ResourcesBeijing, China

**Keywords:** supervisor feedback environment, goal self-concordance, creative performance, creative personality, informal feedback

## Abstract

Studies on the relationship between feedback and creative performance have only focused on the feedback-self and have underestimated the value of the feedback environment. Building on Self Determined Theory, the purpose of this article is to examine the relationship among feedback environment, creative personality, goal self-concordance and creative performance. Hierarchical regression analysis of a sample of 162 supervisor–employee dyads from nine industry firms. The results indicate that supervisor feedback environment is positively related to creative performance, the relationship between the supervisor feedback environment and creative performance is mediated by goal self-concordance perfectly and moderated by creative personality significantly. The mediation effort of goal self-concordance is significantly influenced by creative personality. The implication of improving employees’ creative performance is further discussed. The present study advances several perspectives of previous studies, echoes recent suggestions that organizations interested in stimulating employee creativity might profitably focus on developing work contexts that support it.

## Introduction

The employee’s creative performance is the main source of promoting innovation, enhancing competitiveness, and getting competitive advantage of enterprises (Zhou and Shalley, 2008). Creative performance, defined as the extent to which employees generate novel and useful ideas regarding procedures and processes at work (Baer et al., 2003), has been examined as a function of individual differences, features of the context surrounding employees, and the interaction between the two (Zhou and Shalley, 2008). Creating favorable environments is more likely to get intervention than creative personality and thinking, and the effect of environments can be perceived in short period. Thus creative environment factors become the research hotspot in the field of creativity performance today. Studies have shown that feedback is one of the important environmental factors that influence creative performance (Ford, 1996; Zhou and George, 2001). There are inconsistent results among studies on the relationship between feedback and creative performance. For instance, individuals who receives negative feedback exhibits lower creative performance (Zhou, 1998), but some researchers find that negative feedback is more effective on creative performance than positive feedback (Podsakoff and Farh, 1989). This inconsistency of the research results can lead to the consequence that managers do not know to give employees positive feedback or negative feedback, so solving the inconsistent results feedback effect on creative performance becomes the key point of research (Mulder et al., 2013).

The reasons that researchers conclude about inconsistent results between feedback and creative performance are: first, according to valence, source, and style, feedback is taken a dyadic approach to study its role in creative process, and that it is focused on how isolated feedback interventions impact creative performance (Davidson and De Stobbeleir, 2011) and compare the difference within one dimension. It cannot show the complete picture of feedback (Levy and Williams, 2004; Anseel and Lievens, 2007). This make manager not know how to deliver positive or negative feedback for improving creative performance (Zhou and Shalley, 2008); second, some research ignores feedback receiver’s construction of feedback, which impacts the significance of feedback and then feedback acceptation (Steelman et al., 2004). This lead even manager pay attention to feedback information but cannot get the improvement of creative performance; third, for exploring the mechanism of feedback and creative performance, prior research compares different mechanism between internal and external motivation, this clarification does not conform to the reality that the combination of internal and external motivation influence work outcomes and difficult to explain the inconsistent results about extrinsic motivation and creative performance (Hennessey, 2010); forth, it ignores the individual differences in feedback process and difficult to explain why different employees have different creative performance in the same environment. Thus, it cannot reflect the overall condition of creative performance improving.

In order to fully understand how feedback affects work-related outcomes, researchers should not only consider single feedback interventions, but also consider the broader psychological feedback context in which these feedback interventions take place (Ashford and Northcraft, 2003; Levy and Williams, 2004). Researchers have put forward the concept of the feedback environment, which refers to the contextual processes between the supervisor and subordinate or coworker and coworker in the daily work environment rather than organizing the formal performance appraisal feedback session (Steelman et al., 2004). Feedback environment is a multidimensional structure that can fully reflect feedback content and feedback receiver’s construction of feedback, so it maybe have consistent relationship with creative performance. If the supervisor feedback environment is favorable, feedback will be perceived as information to improve one’s adaption with the target supervisor rather than as an evaluation. This does not mean that favorable feedback environment does not give negative feedback, but rather that the feedback is delivered in a way that does not cause defensiveness. Therefore, feedback receiver can increase trust and result in a more open communication environment in which feedback is used to better align oneself with the target supervisor (Steelman et al., 2004).

With respect to motivation mechanism, in the realistic work environment, purely intrinsically motivated behaviors are limited and rare (Ryan and Deci, 2000). In contrast extrinsically motivated behaviors are be seen everywhere. Therefore, the combination of intrinsic motivation and extrinsic motivation may be able to fully reflect the mechanism of the interference of the feedback environment on creative performance. Goal self-concordance refers to the extent to which activities such as job-related tasks or goals express individuals’ authentic interests and values. Goal self-concordance is one core concept of Self-Determination Theory (SDT), which reveals the effective path of how external interferences influence individual motivation and means that the goal proposed is matched to intrinsic interests and values (Ryan and Deci, 2000). According to SDT, behavior can be chosen freely because of internal or external controls. Thus, individuals’ reasons for acting range on a continuum from complete control to full integration and internalization. There are four types of reasons for engaging in achievement behaviors: “external”(avoid punishment), “introjected” (garner others’ approval), “identified” (achieve a self-valued or personally important goal), and “intrinsic” (experience fun or enjoyment). These reasons form a continuum ranging from external to intrinsic, with the reasons closer on the continuum. Sheldon and Elliot (1999) treated goal self-concordance as a continuum, forming a composite of the two controlled (external and introjected) and two autonomously motivated (identified and intrinsic) reasons for acting. Thus, it would solve the different results of motivation mechanism in relationship between feedback and creative performance.

In regard to individual difference, because employee’s individual characteristics can influence the responses in a particular environment (Tierney et al., 1999; Baer et al., 2003), the one who has individual characteristics about creativity would have more motivation to improve creative performance. It should consider individual characteristics in creative process, the one is creative personality (Sternberg and Lubart, 1996). Sternberg and Lubart (1996) state that individuals with high creative personality can be found following attributes: tolerance of ambiguity, willingness of surmount obstacles and persevere, willingness to grow, sensible risk-taking, belief in oneself. They usually have higher self-confidence, stronger intrinsic motivation, and the resolute desire to be recognized and will work hard to get recognition. So it is necessary to investigate creative personality and feedback environment interaction of goal self-concordance.

The main purpose of this research is to solve these problems by analyzing how feedback environment impacts creative performance via self concordance and analyzing the moderating role of creative personality.

## Theory and Hypotheses

### Supervisor Feedback Environment and Creative Performance

Steelman et al.’s (2004) deem that the feedback environment is composed of seven facets: source credibility, feedback quality, feedback delivery, favorable feedback and unfavorable feedback accurately reflecting performance, source availability, and support for feedback seeking. Source credibility is conceptualized as the feedback source’s expertise and trustworthiness. Consistency and usefulness have been demonstrated to be important aspects of feedback quality. A feedback recipient’s perceptions of the source’s intentions in giving feedback will affect his or her reactions and responses to the feedback. Favorable/unfavorable feedback is conceptualized as the perceived frequency of positive/negative feedback when, from the feedback recipient’s view, his or her performance does in fact warrant positive/negative feedback. Supervisor source availability is operationalized as the perceived amount of contact an employee has with his or her supervisor and/or coworkers and the ease with which feedback can be obtained. Feedback-seeking promotion is defined as the extent to which the environment is supportive or unsupportive of feedback seeking (Rosen et al., 2006). The combination of the facets is thought to reflect a balanced feedback environment. These seven dimensions constitute a highly supportive feedback environment (Whitaker et al., 2007), which could make the employees feel appreciated and cultivate the support of leadership. Such support may motivate employees to approach their work in a more positive way, and increase the salience of the information and the importance of the feedback process (Jennifer and Sabine, 2008). Additionally, with the source of the feedback environment, feedback is further divided into two key feedback sources, coworkers and supervisors. Considering the coworkers and supervisors, the supervisors play a bigger role in influencing creative performance (George and Zhou, 2007). In the current study, we put the supervisor feedback environment to the fundamental position, similar to previous studies (Norris-Watts and Levy, 2004; Anseel and Lievens, 2007). A case can be made for an intimate link between this conceptualization of supervisor supportive feedback environment and creative performance for the following two reasons.

First, the primary literature has shown that some dimensions of the feedback environment influence the employees’ creative performance. For instance, with regard to feedback delivery, research has indicated that if feedback is delivered supportively, then the purpose for informing employees about their contribution is more apparent; thus, the likelihood of improving creative performance is increased (Zhou, 1998). Regarding the validity of feedback, it is important to provide the staff with available and valuable cues to learn, develop and improve performance in the job (Kluger and DeNisi, 1996). However, Steelman et al.’s (2004) definition of favorable and unfavorable feedback provides a precise measurement of the employees’ perception. Based on their research, even veridical shady feedback is positively associated with feedback and with the notion of implementing feedback to improve performance. These results imply that both favorable and unfavorable genuine feedback, with the role of facets of the feedback environment, will stimulate employee creativity. Most literature points out that the quality of feedback is the core dimension of feedback environment.

Second, an upstanding feedback environment is both supportive and incentivizing (Sparr and Sonnentag, 2008), which improves creative performance (Zhou and Shalley, 2008). In supportive feedback environment, employees can better clarify performance standards according to the requirements of organization with less uncertainty and ambiguity (Kim et al., 2010). Concretely speaking, a favorable feedback environment could entitle the employees with the feeling of being appreciated and then cultivate the support of leadership (Sparr and Sonnentag, 2008). Such support may foster and promote employees to approach their work creatively. Indeed, a substantial finding within the creativity literature is the value of a positive and stimulating work environment (Zhou and Shalley, 2008). Consistently, in the laboratory and field research, a positive and stimulating work environment is connected with creativity (Baer et al., 2003), and a non-supportive or a dominant work environment is negatively associated with creativity (Madjar et al., 2002; Zhou, 2003). Such support can originate from a variety of sources, such as supportive leadership (e.g., Shin and Zhou, 2003; Tierney and Farmer, 2004). Considering the above arguments, we offer the following assumption:

Hypothesis 1: Supportive supervisor feedback environment will relate positively to creative performance.

### Supportive Supervisor Feedback Environment and Goal Self-Concordance

In view of the SDT (Ryan and Deci, 2000), goal self-concordance just means the extrinsic motivation degree of internalization. Goal self-concordance refers to the integration extent to which individuals set goals with their intrinsic interest and value, which is usually specified by intrinsic motivation, adding internalized extrinsic motivation and then subtracting controlling extrinsic motivation. This is a new construction of integrating internal motivation and external motivation influences, which helps to resolve the confusing divergence about the relationship between intrinsic motivation and extrinsic motivation. Self-concordance indicates the phenomenon that if employees seek their work goals with a sense of self-determination, rather than with a feeling of obligation, they can achieve their work destination as they identify with these objectives or view these objectives as highly interesting and favorable (Sheldon and Elliot, 1999). Building on the theoretical analysis, two arguments can explain why the supervisor feedback environment influences goal self-concordance.

First, goal self-concordance refers to a broad continuum of goal-based motivations, which integrates the internal and external motivations, assesses the degree to which internalization of work goals arrive, and assimilates the extent of self-goals. SDT acknowledges that an individual’s behavior is not purely motivated by intrinsic elements (Ryan and Deci, 2000), and it is incomplete to study creative performance only from an intrinsic motivation angle. That is to say, goal self-concordance could interpret the motivational mechanism produced by creative performance, and such self-concordance might supply a more realistic and accurate motivational measure than only considering the intrinsic motivation (Davidson and De Stobbeleir, 2011).

Second, SDT suggests that feedback could promote the internalization of employees, followed by goal self-concordance (Ryan and Deci, 2000). The feedback environment could affect the employee goal self-concordance. Informational guides could lead employees to internalize their work goals and assume that these goals are in self-concordance. Employees in the high-level feedback environment express much more feedback seeking need and continuously make valuable decisions in a supportive and friendly communicative way, which develops a sense of ownership at work (Sparr and Sonnentag, 2008). A high-quality feedback environment entitles employees with tactical relevant information to accomplish goal progress, which can foster their intrinsic interest and encourage employees to concentrate their attention on work goals rather than on external worries and concerns (Zhou, 2003). Supportive feedback environment can provide the information and strategies for employees to accomplish work tasks. This environment could increase employees’ interest in work and help them focus more attention on work goals instead of being worried and concerned about other external factors (Steelman et al., 2004).

At last, employees could not only appreciate the messages to finish tasks but also sense its significant meaning in the favorable feedback environment. This type of supportive and encouraging behavior includes the following actions: offering useful suggestions, monitoring the employees’ feelings when receiving supervisor feedback, letting the employees know that their work is acknowledged, and responding rapidly (Steelman et al., 2004). In short, a constructive and informative feedback environment advances the internalization of work goals (Zhou and George, 2001).

Hypothesis 2: Supportive supervisor feedback environment will relate positively to goal self-concordance.

### The Mediating Role of Goal Self-Concordance

Previous studies have shown that employees’ level of goal self-concordance is related to their happiness, job satisfaction, and performance (Sheldon and Elliot, 1999; Sheldon and Houser-Marko, 2001; Bono and Judge, 2003; Miquelon and Vallerand, 2008). Bono and Judge (2003) found that the more self-concordant work goals individuals demonstrate, the more initiative the work represents and the better the creative performance is. Building on this theoretical analysis, two arguments can explain why self-concordance affects creative performance.

First, as Sheldon and Elliot (1999) noted, a core characteristic of self-concordance is that employees feel ownership in their goals. Self-concordant goals express employees’ developing interests and intrinsic values; thus, employees feel responsible for these goals and are stimulated to spare no efforts to pursue their goals (Sheldon and Elliot, 1999; Ryan and Deci, 2000). Therefore, individuals who continuously strive for self-concordant goals are more likely to show solicitude for their goals and spare no efforts in pursuing them, which are two preconditions for creative performance (Zhou and Shalley, 2008).

Furthermore, it is easier for individuals to find some alternatives to solve problems when they pursue self-concordant work goals, such as using non-traditional approaches to solve their work problems, which leads to more creativity (Zhou and Shalley, 2008). For example, research has shown that individuals who pursue goals that are consistent with their interior interests and values are able to be more flexible, while individuals who display more external reasons in work activity tend to take on more rigid cognition (Deci and Ryan, 1987). On the basis of these arguments, we suppose the following:

Building on the arguments for the above two assumptions and the empirical evidence about how self-concordance affects creative performance in contextual factors (Bono and Judge, 2003), goal self-concordance could play a mediate role when the supervisor feedback environment influences creative performance. It is perceived that when employees apperceive their supervisors to offer them useful, credible and supportive responses to feedback-seeking behavior, they are inclined to be more responsible and take ownership of the targets. Improving goal self-concordance could stimulate individuals to strive for work and be more flexible to cognize work, which will lead to individuals exhibiting superior creative performance.

Hypothesis 3: Goal self-concordance mediates the relationship between employees’ perceptions of the supervisor feedback environment and creative performance.

### The Moderating Role of Creative Personality

According to Field theory, behavior is a function of the individual and the psychological environment, and the mechanism of how the feedback environment impacts creative performance should consider individual differences. Creative personality is an integrated structure that unifies intelligence factors and non-intellectual factors, which determines people’s creativity. Prior research has shown that creative personality and context factors interact to influence the creative performance of the staff (Oldham and Cummings, 1996; Zhou and George, 2001). Oldham and Cummings (1996) examine the mutual influence of employees’ creativity and creative performance in the two characteristics of the organizational context, which are job complexity and supportive supervision, with the conclusion that the employees represent the maximum creativity when taking on complex and challenging assignments with supportive and non-controlling supervision. George and Zhou (2001) found that participants with superior patent personality display a high-level creativity after receiving positive leadership feedback and luminous tasks, and job holders with inferior creative personality perform at a higher-level of creativity if the executive behavior is less controlling. The above results indicate that the personality of the staff influences their reaction to social environmental factors, and the diverse environmental features interact with the employees’ creativity.

Sternberg and Lubart (1996) state that individuals with high creative personality can adapt to uncertain situations; as they encounter difficulties, they can assume a reasonable level of risk and continue to progress. They usually have higher self-confidence, stronger intrinsic motivation, and the resolute desire to be recognized and will work hard to get recognition (Sternberg and Lubart, 1996). A supportive work environment makes employees feel valued and as if they are taken seriously by their supervisor (Sparr and Sonnentag, 2008). Research on the role of feedback on creativity has already shown that creative personality is an important moderator of the feedback-creativity relationship. For example, Zhou and Shalley (2008) showed developmental feedback and creative personality on the creative performance. The highest creative performances were exhibited when individuals receive developmental feedback and had a creative personality. In addition, in the feedback literature, Ilgen et al. (1979) argue that, apart from feedback, personality is an important condition to promote internalization of work assignments. For creative performance, creative personality would predict creativity through intrinsic motivation in combination with other factors such as social support in a complex way but which could be modeled (Sternberg and Lubart, 1996). Therefore, for employees with a high creative personality, a supportive feedback environment would meet the needs of the desire to be recognized and will further promote effort to do more work. Basic psychological needs theory suggests that when environmental factors fulfill psychological needs, the internalization of intrinsic motivation and extrinsic motivation will be promoted, thus contributing to the behavior of individuals.

Hypothesis 4: creative personality will moderate the strength of the mediated relationship between supervisor feedback environment and creative performance via goal self-concordance, such that the relationship will be stronger when employee is high creative personality than low.

**FIGURE 1:**
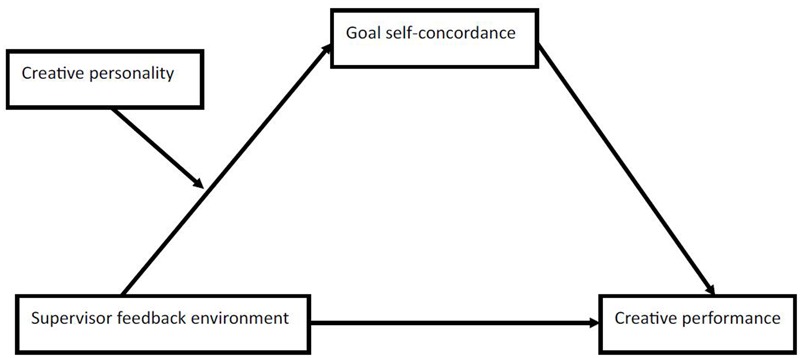
**Test model**.

We have developed moderated mediation hypotheses for supervisor feedback environment and build up our research model (**Figure 1**).

## Materials and Methods

### Participants and Procedures

A convenience sampling of 172 supervisor-subordinate dyads from nine industrial enterprises based in Beijing, China. The entire sample is Chinese. The firm’s HR manager helped collect the data by preparing a list of randomly selected employees (172) and their supervisors (25), on average one supervisor evaluated nearly seven employees. Employees and their supervisors provided data on separate questionnaires and on different occasions.

To avoid common method bias, data were collected from two sources: the employees and their supervisors. The employees filled out a questionnaire that included items measuring the independent variables, mediation variables, demographics, his or her name and supervisor’s name. In a separate questionnaire, each employee’s supervisor rated the employee’s creativity. The supervisors assigned to complete the rating forms were those who had plenty of opportunity to observe their employees’ creative performance.

We assigned an identification number to each employee and his or her supervisors to match the employee questionnaires with the responses of their supervisors. They completed the questionnaires during their work hours and then returned them. One hundred and sixty-two usable dyads were returned, giving a response rate of 94%. Demographic information indicated that there were 95 male employees (58.6%) and 67 female managers (41.4%) in the sample. More than half of the employees were 20-30 years of age, 95% of the participants were under 40 years old; 56% of the participants held a bachelor’s degree or above; 63% of the participants worked for less than 3 years, 81% had worked for less than 5 years.

### Ethics statement

This study was reviewed and approved by of American Psychological Association Ethics Committee Rules and Procedures, APA Ethics Committee with written informed consent from all participants. All participants gave written informed consent in accordance with the Declaration of Helsinki.

### Instruments

The measure items used in the present study were primarily developed in English; thus, to ensure cross-linguistic equivalence, we translated all scale items into Chinese and then translated them back into English by means of two bilingual (English–Chinese) professional translators (Brislin, 1980).

#### Supervisor Feedback Environment

We measured the supervisor feedback environment using Steelman et al.’s (2004) scale. This Likert scale assesses each feedback environment dimension and the seven facets within each dimension. A sample item from the Source Credibility scale reads, “my supervisor is generally familiar with my performance on the job.” Feedback Quality was measured with items such as “my supervisor gives me useful feedback about my job performance.” An example from the Feedback Delivery scale reads “my supervisor is supportive when giving me feedback about my job performance.” An item from the Favorable Feedback scale is “my supervisor generally lets me know when I do a good job at work,” while the Unfavorable Feedback scale includes items such as “my supervisor tells me when my work performance does not meet organizational standards.” An example of an item from the Source Availability scale reads “my supervisor is usually available when I want performance information.” The Promotes Feedback Seeking scale includes items such as “my supervisor encourages me to ask for feedback whenever I am uncertain about my job performance.” Because the hypotheses in this study operate at the construct level, our analyses used a composite score of the feedback environment rather than a score based on the individual facets. The Cronbach’s α for the measure of supervisor feedback environment was 0.73.

#### Goal Self-Concordance

We measured goal self-concordance by using Deci and Ryan’s (1987) scale. The statements such as “You choose this goal because somebody else wants you to or because the situation demands it” and “You pursue this goal because you would feel anxious, guilty, or ashamed if you didn’t” (external and introjected items represent controlled motivation, each of these checked adjectives was given a value of -1); “You pursue this goal because you really believe it’s an important goal to have” and “You pursue this goal because of the fun and enjoyment it provides you” (identified and intrinsic items represent autonomous motivation, each of these checked adjectives was given a value of +1). The values were then summed to calculate goal self-concordance. The Cronbach’s α for the measure of goal self-concordance was 0.86.

### Creative Personality

The 30-item Creative Personality Scale (Gough, 1979) of the Adjective Check List (ACL) (Gough and Heilbrun, 1965) was used to assess employees’ creativity-relevant personal characteristics. Employees were asked to “place a check mark next to each adjective that you think describes you.” Of the 30 adjectives, 18 describe highly creative people: capable, clever, confident, egotistical, humorous, informal, individualistic, insightful, intelligent, interests wide, inventive, original, reflective, resourceful, self-confident, sexy, snobbish, and unconventional. Each of these checked adjectives was given a value of +1. The remaining 12 adjectives describe less creative people: cautious, commonplace, conservative, conventional, dissatisfied, honest, interests narrow, mannerly, sincere, submissive, suspicious, and phony. Each of these checked adjectives was assigned a value of -1. The values were then summed to form a creative personality index. The Cronbach’s α for the measure of creative personality was 0.70.

### Creative performance

Consistent with prior studies, we used supervisor ratings to assess employees’ creative performance (Zhou, 1998; Zhou and George, 2001). Using 13 items each supervisor rated how often their subordinates displayed creative behavior in the workplace on a 5-point scale ranging from never to always. A sample item taken from the scale reads “Seeks out new technologies, processes, techniques and/or product ideas.” The Cronbach’s α for the measure of creative performance was 0.75.

### Controls

Consistent with previous creativity research (e.g., Zhou and George, 2001; Shalley et al., 2004), we controlled for demographic variables that have been found to be significantly related to creativity: age, gender, job tenure, and education.

## Results

To examine the convergent and discriminant validity of the key variables, we employed structural equation modeling to conduct the discrimination validity of confirmatory factor analysis (CFA) using AMOS 21.0. We assessed overall model fit by goodness-of-fit indices including the goodness fit index (GFI), normalized fit index (NFI), relative fit index (RFI), comparative fit index (CFI), incremental fit index (IFI), and root mean square error of approximation (RMSEA). A reasonable model fit is indicated when the CFI and IFI are above 0.90 and the RMSEA is below 0.08 (Hu and Bentler, 1998). According to Wang et al.’s (2005) method for testing the discrimination validity of the CFA, we compared a four-factor model (Model 1) with two three-factor models (Model 2 and Model 3), a two-factor model (Model 4), and a one-factor model (Model 5). In the four-factor model, we treated four constructs (supervisor feedback environment, goal self-concordance, creative personality, and creative performance) as four independent factors. In the first three-factor model (Model 2), we loaded supervisor feedback environment and creative performance items on one factor. In the second three-factor model (Model 3), we loaded goal self-concordance and creative personality items on one factor. In the two-factor model (Model 4), we loaded goal self-concordance, creative personality, and creative performance items on one factor. In the one-factor model (Model 5), we loaded all variables on one factor. The result shows that the four-factor model fits the data better than other nested models (see **Table 1**), indicating that the four variables show a good discriminant validity. In summary, the CFA results suggest that the respondents could distinguish clearly the constructs under study.

**Table 1:** Confirmatory factor analysis of discrimination validity.

Model	Factor loaded	χ^2^/df	GFI	NFI	RFI	IFI	CFI	RMSEA
Model 1	Four factors: SFE, GSC, CPS, CP	2.19	0.98	0.97	0.97	0.98	0.98	0.06
Model 2	Three factors: SFE and CP are combined into one factor	2.47	0.76	0.75	0.71	0.76	0.76	0.09
Model 3	Three factors: GSC and CPS are combined into one factor	2.23	0.84	0.84	0.80	0.85	0.85	0.09
Model 4	Two factors: GSC, CPS, CP are combined into one factor	2.47	0.71	0.70	0.70	0.71	0.70	0.1
Model 5	One factor: all variables are combined into one factor	2.63	0.48	0.46	0.46	0.48	0.48	0.11

*n = 162. SFE, supervisor feedback environment; GSC, goal self-concordance; CPS, creative personality; CP, creative performance.*

**Table 2** presents the means, standard deviations, and correlations among the study variables. An inspection of the correlations reveals that the supervisor feedback environment (*r* = 0.37, *p* < 0.01) is positively related to self-concordance and also positively related to creative performance (*r* = 0.20, *p* < 0.01). The results also indicate that goal self-concordance is positively related to creative performance (*r* = 0.29, *p* < 0.01) and also positively related to creative personality (*r* = 0.31, *p* < 0.01). In addition, creative personality related positively to goal self-concordance (*r* = 0.31, *p* < 0.01) and creative performance (*r* = 0.17, *p* < 0.01). The results of correlation analysis generally supported the positive effects of supervisor feedback environment on creative performance.

**Table 2:** Means, standard deviations, and correlations of all measures.

	Mean	*SD*	1	2	3	4	5	6	7	8
1. Gender	1.41	0.49	-							
2. Age	1.50	0.58	0.01	-						
3. Education	1.88	0.57	0.32**	0.19*	-					
4. Tenure	2.41	1.26	0.06	0.44**	0.16	-				
5. SFE	4.82	0.50	-0.2*	-0.16*	-0.13	-0.14^∗∗^	-			
6. GSC	0.55	0.71	-0.16*	-0.21**	-0.34*	-0.3^∗∗^	0.37^∗∗^	-		
7. CPS	2.90	3.79	-0.33**	0.01	-0.22**	-0.16	0.13	0.31^∗∗^	-	
8. CP	3.46	0.32	0.08	-0.05	0.36**	-0.17	0.2^∗∗^	0.29^∗∗^	0.17^∗∗^	-

*n = 162; ^∗^*p* < 0.05, ^∗∗^*p* < 0.01. SFE, supervisor feedback environment; GSC, goal self-concordance; CPS, creative personality; CP, creative performance.*

Further analyses were conducted to better estimate the overall contribution of supervisor feedback environment in predicting outcome variables as well as the mediation role of goal self-concordance. To examine whether goal self-concordance served as a mediator for the relations between supervisor feedback environment and creative performance, we adopted the procedure proposed by Preacher and Hayes (2008). According to their suggestions, there are three criteria to justify a mediation effect. First, the independent variable should be significantly correlated with mediator variable. Second, after the effect of the independent variable toward dependent variable was controlled, the correlation between mediator variable and dependent variable should be significant. Finally, the indirect effect of independent variable on dependent variable must be significant. Before the analyses, all continuous predictors were well-centered (Aiken et al., 1991). As showed in **Table 3**, after controlling for the effect of participants’ demographics (gender, age, education, and tenure), supervisor feedback environment significantly predicted goal self-concordance (Model 1: β = 0.29, *p* < 0.01).

**Table 3:** Hierarchical regressions for the impact of supervisor feedback environment and self-concordance on creative performance.

	Goal self-concordance as dependent variable		Creative performance as dependent variable	
	Model 1	Model 2	Model 3	Model 4
Gender	-0.01	0.07	-0.01	-0.01
Age	-0.03	-0.06	-0.01	0.01
Education	-0.26	-0.23	-0.17	-0.11
Tenure	-0.20	-0.16	0.05	0.1
Supervisor feedback environment	0.29**	0.27**	0.23**	0.16
Goal self-concordance				0.24**
Creative personality		0.23**		
Supervisor feedback environment × creative personality		0.14*		
*R*^2^	0.25	0.32	0.09	0.13
Δ*R*^2^	0.23	0.07	0.06	0.1
*F*	9.43**	10.36**	2.93*	3.81**

*n = 162; ^∗^*p* < 0.05, ^∗∗^*p* < 0.01; values are standardized coefficients.*

Logistic regression was conducted to predict creative performance. As showed in **Table 2**, supervisor feedback environment severed as the significant predictor of creative performance (Model 3:β = 0.23, *p* < 0.01). When adding goal self-concordance to the model, goal self-concordance also significantly predicted employment status career adaptability (Model 4: β = 0.24, *p* < 0.01), but the effect of the supervisor feedback environment on creative performance (Model 3: β = 0.16, ns) became non-significant. To calculate the indirect effects, we adopted the SPSS micro PROCESS (Hayes, 2013). Results in **Table 4** show that the formal two-tailed significance test (assuming a normal distribution) demonstrated that the indirect effect was significant (Sobel *z* = 2.59, *p* < 0.01). Bootstrap results confirmed the Sobel test, with bootstrap 95% confidence interval of 0.02–0.12 around the indirect effect not containing 0. Taken together, Hypothesis 1-3 received full support.

**Table 4:** Results of Sobel test and bootstrap for the indirect effect of supervisor feedback environment on creative performance via goal self-concordance.

	Value	*SE*	*z*	*p*	LL 95% CI	UL 95% CI
Sobel test results for indirect effect	0.06	0.02	2.59	0.00	0.02	0.13
Bootstrap results for indirect effect	0.06	0.02	2.60	0.00	0.02	0.12

*n = 162. LL, lower limit; CI, confidence interval; UL, upper limit.*

To test the moderated mediation (Hypothesis 4), we examined four conditions (Muller et al., 2005; Preacher et al., 2007): (a) significant effect of supervisor feedback environment on creative performance; (b) significant interaction between supervisor feedback environment and creative personality in predicting goal self-concordance; (c) significant effect of goal self-concordance on creative performance; and (d) different conditional indirect effect of supervisor feedback environment on creative personality, via goal self-concordance, across low and high levels of creative personality.

Our results for Hypothesis 1, which demonstrated that supervisor feedback environment was significantly related to creative performance, supported Condition 1 for moderated mediation. To test for Condition 2, we first examined whether the interaction of supervisor feedback environment with creative personality was significant in predicting goal self-concordance. **Table 3** presents moderated regressions results of creative personality on goal self-concordance and creative performance. It shows that the interaction term for supervisor feedback environment with creative personality was significant in predicting goal self-concordance (Model 2: β = 0.17, *p* < 0.05). To further understand the moderating effect, we plotted the interaction effect using one standard deviation above and below the mean of creative personality (Aiken et al., 1991). **Figure 1** shows the interaction patterns as expected in that the relationship between supervisor feedback environment and goal self-concordance is stronger for low creative personality than high creative personality. Hence, this satisfied Condition 2.

**FIGURE 2:**
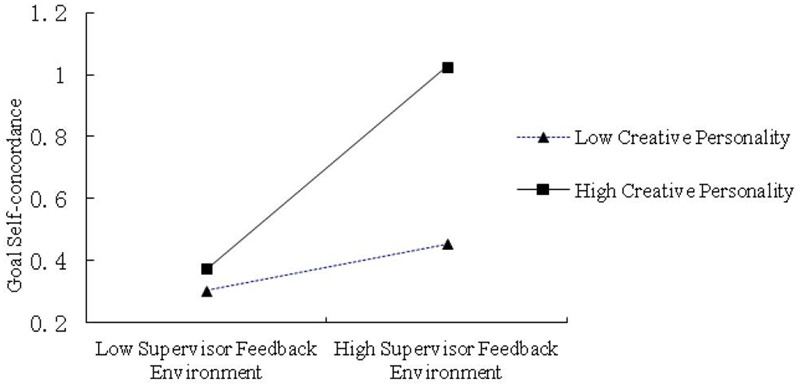
**Simple slopes of supervisor feedback environment predicting goal self-concordance at low (one SD below *M*) and high (one SD above *M*) levels of creative personality**.

The results for Hypothesis 3 supported Condition 3, in which goal self-concordance was positively related to creative performance. Hence, results based on the first three conditions indicate that creative personality could moderate the mediation of goal self-concordance for the supervisor feedback environment–creative performance association. **Figure 2** shows that the interaction patterns were as expected, in that the relationship between supervisor feedback environment and goal self-concordance was stronger for the employee who has high creative personality (1 standard deviation above the mean) than low creative personality (1 standard deviation below the mean).

To further validate findings of moderated mediation relationships, we used Preacher et al.’s (2007) statistical significance test, to compute a *z* statistic for the conditional indirect effect (Condition 4). More specifically, we operationalized high and low levels of creative personality as one standard deviation above, moderate level and below the variable’s mean score. Results in **Table 5** show that the conditional indirect effect of supervisor feedback environment was stronger and significant in the high creative personality (conditional indirect effect = 0.65, *SE* = 0.15, *z* = 4.5, *p* < 0.01, with 95% confidence interval of 0.37–0.94) and in moderate level of creative personality (conditional indirect effect = 0.4, *SE* = 0.10, *z* = 4.04, *p* < 0.01, with 95% confidence interval of 0.20–0.59), but was stronger and not significant in the low creative personality condition (conditional indirect effect = 0.14, *SE* = .15, *z* = 0.93, ns, with 95% confidence interval of -0.16 to 0.44). Taken together, Hypothesis 4 was supported.

**Table 5:** Results for conditional indirect effect of supervisor feedback environment on creative performance via goal self-concordance across levels of creative personality.

Moderator level	Mean	Conditional indirect effect	*SE*	*z*	*p*	LL 95% CI	UL 95% CI
Low (M -1 SD)	-0.38	0.14	0.15	0.93	0.35	-0.16	0.44
Moderate level	0	0.4	0.10	4.04	0.00	0.20	0.59
High (M +1 SD)	0.38	0.65	0.15	4.50	0.00	0.37	0.94

## Discussion

The present study contributes to the literature on the feedback-creativity relationship by highlighting employees’ perceptions of the broader feedback environment as a new avenue for enhancing employee creative performance. Our results show that employees’ perceptions of a supportive supervisor feedback environment indirectly influence their level of creative performance through the internalization of work goals. In addition, we indicate that creative personality moderates this mediation, such that the more creative personality employees are at work, the stronger this relationship is.

Regarding relevant research on supervisor feedback environment, a positive supervisor feedback environment provides useful information to help employees in completing their work (Zhou and Shalley, 2008). Therefore, a supportive leadership feedback environment will have a positive role in promoting creative performance, which is consistent with the previous theoretical analyses and studies (Steelman et al., 2004; Davidson and De Stobbeleir, 2011). The shift of the present study toward feedback is in line with a recent trend within the feedback literature, which increasingly looks at the broader psychological feedback context in which feedback interventions take place (Sparr and Sonnentag, 2008; Kim et al., 2010). The finding that a supportive feedback environment is a way to enhance employee creative performance fits well with prior research demonstrating the importance of a supportive environment for creative performance (Madjar et al., 2002; Zhou, 2003). Supportive supervisor feedback environment lets employees feel as if leaders pay more attention to their work, thus promoting their feedback seeking behavior. This behavior will further help employees be more focused on content and generate interest, with employees interested in their work and their goals as goal self-concordance (Davidson and De Stobbeleir, 2011), to make employees internalize their objectives (Ryan and Deci, 2000; Gagné and Deci, 2005), which contributes to the generation of creative performance. A previous study has found that personality is related to creative performance, and openness to experience had positive relationship with creative performance (Oldham and Cummings, 1996). Also, SDT proposes that social environment, which can be characterized as autonomy supportive, can fulfill the basic psychological needs for competence, relatedness, and autonomy. These basic psychological needs fulfilling will support intrinsic motivation and facilitate internalization of extrinsic motivation, and make one’s goal matched to intrinsic interests and values which is same like goal self-concordance (Ryan and Deci, 2000). This will in turn yield the important work outcomes of (1) persistence and maintained behavior change; (2) effective performance, particularly on tasks requiring creativity, cognitive flexibility, and conceptual understanding; (3) positive work-related attitudes (Ryan and Deci, 2000; Gagné and Deci, 2005). Our results for mediating role of goal self-concordance demonstrate this path too. Individuals with a high creative personality are easily affected by the supervisor feedback environment. These individuals can then make rapid adjustments in response to the feedback content to pursue a positive behavior, to cater to the change of external environment, and to improve creative performance (Zhou, 2003). Employees with creative personalities like challenging work, which stimulates their intrinsic motivation and promotes creative performance. In SDT, regarding the integration of individual differences, extrinsic motivation not only depends on environmental factors but also is subject to an individual’s internal resources as to the degree of internalization in a particular situation. The social environment impact on the individual consolidation process exhibits a significant difference (Deci et al., 1994). Oldham and Cummings (1996) stated that individuals are most creative when taking on complex and challenging work, and one’s intrinsic motivation influences creative performance in a supportive but not dominant guidance environment. This study verified the above scholars’ point of view from an empirical angle, providing strong empirical data for the effects that creative personality and environmental factors have on the creative performance.

### Theoretical Contribution

The present study advances several perspectives of previous studies. First, our exploration of the impact on burnout of feedback as a multi-dimensional variable helps resolve the inconsistencies of previous studies resulting from only considering feedback from single dimension. Our assumption that employees’ perceptions of the expanded feedback environment indirectly influence their creative performance emphasizes the relevance of studying more aggregate psychological feedback context within the creativity domain. Traditionally, the literature on the feedback-creativity relationship has focused on the effect of specific feedback components, such as feedback valence and feedback delivery, on creative performance (Zhou, 1998, 2003; George and Zhou, 2001; Zhou and George, 2001). The shift of the present study toward a more comprehensive conceptualization of feedback is in line with a recent trend within the feedback literature, which increasingly looks at the broader psychological feedback context in which feedback interventions take place (e.g., Herold and Parsons, 1985; Ashford and Northcraft, 2003; Levy and Williams, 2004; Steelman et al., 2004; Anseel and Lievens, 2007).

Second, our results add to the creativity literature by identifying goal self-concordance as a mediating mechanism between perceptions of a favorable supervisor feedback environment and creative performance. While prior theorizing within the creativity and the feedback–creativity domain has focused on intrinsic motivation as a motivational mechanism between contextual factors and creative performance (Shalley et al., 2004), empirical research on the mediating role of intrinsic motivation has yielded inconsistent results (Shalley and Perry-Smith, 2001; Shin and Zhou, 2003). In response to these inconsistent results, Shalley et al. (2004) suggested that contextual characteristics may not only affect creativity via intrinsic motivation but also via a number of alternative mechanisms. The present study indicates that goal self-concordance, which provides a more balanced view on motivation, can be such an additional mechanism. Our findings provides further support for Bono and Judge’s (2003) findings that contextual factors, such as a favorable feedback environment, influence the extent to which individuals perceive their work activities to be aligned with their authentic interests and values, and that when individuals do have such perceptions, they are likely to display a higher level of creativity throughout their goal striving.

Third, by introducing creative personality as a moderator of the mediated feedback-creativity relationship, this study sheds light on an important boundary condition that strengthens the relationship between feedback and creativity. While these findings are in line with previous research by George and Zhou (2001) our model extends beyond that study, in that we examined creative personality as a moderator to the mediated relationship between supervisor feedback environment, goal self-concordance and creative performance rather than as a moderator to the direct relationship between feedback and creativity. As hypothesized, the highest level of goal self-concordance was found for the supportive feedback environment and high creative personality condition, but for employee who has low level of creative personality this effect is not significant. This finding reveals that certain types of employees are more susceptible to supervisor feedback environment. Moreover, the study’s moderated mediation results have underscored the importance of creating supportive supervisor feedback environment for employee who has creative personality. Our research efforts might further prior understand with regard to the factors that impact creative performance by including relevant additional variables. Some researchers are considering the increased interest in the interplay of personal and contextual characteristics (Shalley et al., 2004; Zhou and Shalley, 2008), they think it might be valuable to investigate how personal characteristics relevant to creativity, such as personality, cognitive style and creative role identity, interact with perceptions of the feedback environment. Indeed, prior research suggests that personal characteristics do influence the way individuals respond to contextual factors (Tierney et al., 1999; Baer et al., 2003). Somewhat surprisingly, however, the conditional indirect effect of supervisor feedback environment was stronger and significant in the high creative personality and in moderate level of creative personality, but was stronger and not significant in the low creative personality condition. In order to explain this finding, we build on previous theoretical suggestions (e.g., Ilgen et al., 1979) in proposing that even high-quality feedback considerately delivered by credible and available supervisors might not satisfy their psychological need and catch goal self-concordance if employees do not have creative personality. This lack of creative personality might in fact not only prevent employees from taking ownership of their goals and integrate them into themselves, but this might also create frustration and increases the feeling that one lacks personal psychological need satisfaction resulting in lower levels of self-concordance.

Finally, The results also have some contribution to SDT. SDT proposes that people who are high in the autonomous causality orientation tend to be more autonomously motivated in a particular situation and to show positive performance and well-being outcomes, but rarely research test whether this orientation would influence creative performance through goal self-concordance. Different goals need different trains. In the creativity literature, Zhou and Shalley (2008) found creative personality correlated with creative performance. In the present research, we built upon this earlier literature and found creative personality can influence goal self-concordance and then influence creative performance, like Oldham and Cummings (1996) found that creative performance was highest when employee had appropriate creativity-relevant characteristics and worked in complex jobs with supportive and no controlling supervision. Individuals with high creative personality might be identified through use of assessment instruments (Gough, 1979) such as the feedback environment and the normative baselines that accompany these instruments. They can find more meaning and interest from feedback environment and fulfill their psychological needs, the internalization of intrinsic motivation and extrinsic motivation will be promoted, thus contributing to the creative behavior of individuals if feedback environment is supportive (Ryan and Deci, 2000).

### Practical Contribution

Our study echoes recent suggestions that organizations interested in stimulating employee creativity might profitably focus on developing work contexts that support it. As discussed by Shalley and Zhou (2008), such contexts may be developed by setting creativity goals, making creativity a job requirement and building reward systems that value employee creativity. Based on this study, Leaders should strive to build a supportive feedback environment, improve feedback credibility, have support staff actively seeking feedback behavior, improve the quality of their feedback and offer feedback with taking more consideration of employees’ psychological experience, etc. Organizations may develop contexts that support creativity by training employees to give each other well-construed feedback and encouraging them to seek feedback from each other, rather than limiting themselves to supervisor-delivered feedback.

Our results also suggest that if creative performance is the organization’s goal, there is value in stimulating effective feedback exchanges by focusing on the supervisor–employee relationship. In the enterprise management practice, it is essential not only to achieve flow of top-down feedback but also ensure the feedback in parallel communication. In the enterprise practice, if an employee needs complete creative task, the supervisor can choose an employee who has a high creative personality.

### Limitations and Future Research Suggestions

Although our findings have made some contribution to answering several recent questions in the feedback-creative performance literature, this study has several limitations.

One limitation is the sample. The small sample size reported here may have affected the current results. However, this small sample size coupled with the significant results suggests that the current findings are reliable. our data were from an organization in China, and therefore the external validity of our findings may not be accurate. Our analysis does not preclude different interpretations in other settings since organizational or cultural difference may influence employees’ attitudes and behaviors. Future studies should add more samples, which will make the results more specific and representative. To improve the generalizability of our results, future research should replicate our model employing multi-organization and cross-national samples.

Second, this study relies on cross-sectional data; therefore, no causal relationships of the studied variables can be unambiguously established, because the longitudinal research design may have other explanations. It may be that some of the relationships we found may operate in reverse. For example, it may be that a favorable feedback environment does not promote internalization of work goals, but that when employees pursue self-concordant work goals and thus demonstrate high levels of ownership with regard to their work goals, supervisors are more likely to provide them with well-construed and high-quality feedback and to be receptive and available when these employees seek feedback. To ascertain causality, future studies can start from similar experimental designs or from time-series designs to collect longitudinal data that clarifies the causal relationships among the studied variables.

Third, prior studies have demonstrate that supervisors engaged in close monitoring and unsupportive coworkers (Zhou and George, 2001), rewarding creativity (Sue-Chan and Hempel, 2016), and task autonomy (Davidson and De Stobbeleir, 2011) can influence creative performance, but in this research we only focused on demographic variables as control variables. Future research should add more diverse control variables for a deeper understanding of relevant issues and add more confidence to the conclusions.

Last, the coworker feedback environment is also very important. In a work field where employees usually have a lot of contact with coworkers, the coworkers’ feedback environment may play a different role in affecting creative performance. By incorporating coworkers as well as supervisors as important feedback sources, some study recognized and found support for the importance of coworker feedback with regard to creative performance. By showing that employees’ perceptions of a favorable coworker feedback environment stimulate them to be creative throughout their goal striving, in future not only we should focus on supervisor feedback and feedback as a generic construct (Zhou and George, 2001; George and Zhou, 2007), but also highlight the importance of coworkers in the workplace (Chiaburu and Harrison, 2008).

## Author Contributions

JZ provided substantial contributions to the research conception and design. ZG and SZ analyzed and interpreted the data. ZG, SZ, and YZ wrote the paper, JZ provided critical revisions of the paper. ZG and SZ both attended to the revision of the paper. JZ and ZG both approved of this version of the paper to be published.

## Conflict of Interest Statement

The authors declare that the research was conducted in the absence of any commercial or financial relationships that could be construed as a potential conflict of interest.
